# Resuscitation With Vitamin C, Hydrocortisone, and Thiamin in Children With Septic Shock: A Multicenter Randomized Pilot Study

**DOI:** 10.1097/PCC.0000000000003346

**Published:** 2024-01-19

**Authors:** Luregn J. Schlapbach, Sainath Raman, David Buckley, Shane George, Megan King, Roberta Ridolfi, Amanda Harley, Michele Cree, Debbie Long, Simon Erickson, Puneet Singh, Marino Festa, Kristen Gibbons, Rinaldo Bellomo

**Affiliations:** 1 Child Health Research Centre, The University of Queensland, Brisbane, QLD, Australia.; 2 Paediatric Intensive Care Unit, Queensland Children’s Hospital, Children’s Health Queensland, Brisbane, QLD, Australia.; 3 Department of Intensive Care and Neonatology, and Children’s Research Center, University Children’s Hospital Zurich, University of Zurich, Zurich, Switzerland.; 4 Paediatric Intensive Care Unit, Starship Children’s Hospital, Auckland, New Zealand.; 5 Departments of Emergency Medicine and Children’s Critical Care, Gold Coast University Hospital, Southport, QLD, Australia.; 6 School of Medicine and Menzies Health Institute Queensland, Griffith University, Southport, QLD, Australia.; 7 School of Nursing, Midwifery and Social Work, University of Queensland, QLD, Australia.; 8 School of Nursing, Centre for Healthcare Transformation, Queensland University of Technology, Brisbane, QLD, Australia.; 9 Paediatric Critical Care Unit, Perth Children`s Hospital, Perth, WA, Australia.; 10 Paediatric Intensive Care Unit, Sydney Children’s Hospital, Sydney, NSW, Australia.; 11 Paediatric Intensive Care Unit, Children’s Hospital Westmead, Sydney, NSW, Australia.; 12 Kids Critical Care Research Group, Kids Research, Sydney Children’s Hospitals Network, Sydney, NSW, Australia.; 13 Intensive Care Research, Austin Hospital and Monash University, Melbourne, VIC, Australia.; 14 Department of Critical Care, University of Melbourne., Melbourne, VIC, Australia.; 15 Australian and New Zealand Research Centre, Monash University, Melbourne, VIC, Australia.; 16 Department of Intensive Care, Royal Melbourne Hospital, Melbourne, VIC, Australia.; Starship Children’s Hospital; Perth Children`s Hospital; Queensland Children’s Hospital and The University of Queensland; Gold Coast University Hospital; Sydney Children’s Hospital; Royal Children’s Hospital; Monash Children`s Hospital; Women’s and Children’s Hospital; Children’s Hospital Westmead, Sydney, Australia

**Keywords:** ascorbic acid, child, hydrocortisone, inotropes, intensive care, resuscitation, sepsis, septic shock, thiamin, vitamin C

## Abstract

**OBJECTIVES:**

Adjunctive therapy with vitamin C, hydrocortisone, and thiamin has been evaluated in adults, but randomized controlled trial (RCT) data in children are lacking. We aimed to test the feasibility of vitamin C, hydrocortisone, and thiamin in PICU patients with septic shock; and to explore whether the intervention is associated with increased survival free of organ dysfunction.

**DESIGN:**

Open-label parallel, pilot RCT multicenter study. The primary endpoint was feasibility. Clinical endpoints included survival free of organ dysfunction censored at 28 days and nine secondary outcomes, shock reversal, and two proxy measures of intervention efficacy.

**SETTING:**

Six PICUs in Australia and New Zealand.

**PATIENTS:**

Children of age between 28 days and 18 years requiring vasoactive drugs for septic shock between August 2019 and March 2021.

**INTERVENTIONS:**

Patients were assigned 1:1 to receive 1 mg/kg hydrocortisone every 6 hours (q6h), 30 mg/kg ascorbic acid q6h, and 4 mg/kg thiamin every 12 hours (*n* = 27), or standard septic shock management (*n* = 33).

**MEASUREMENTS AND MAIN RESULTS:**

Sixty of 77 (78%) eligible patients consented with 91% of approached parents providing consent. The median time from randomization to intervention was 44 (interquartile range [IQR] 29–120) min. Seventy of seventy-seven (28%) patients had received IV steroids before randomization. Median survival alive and free of organ dysfunction was 20.0 (0.0–26.0) days in the intervention and 21.0 (0.0–25.0) days in the standard care group. Median PICU length of stay was 5.3 (2.5–11.3) days in the intervention group versus 6.9 (3.0–11.5) days in the control group. Shock reversal occurred at a median of 35.2 (14.6–101.2) hours in the intervention group versus 47.3 (22.4–106.8) hours in the standard care group (median difference –12 hr; 95% CI, –56.8 to 32.7 hr).

**CONCLUSIONS:**

In children requiring vasopressors for septic shock, a protocol comparing adjunctive treatment with high-dose vitamin C, hydrocortisone, and thiamin versus standard care was feasible. These findings assist in making modifications to the trial protocol to enable a better-designed larger RCT.

**RESEARCH IN CONTEXT**

- Hydrocortisone, ascorbic acid, and thiamin (HAT) have been evaluated extensively as adjunctive sepsis therapy in adults, but randomized data on children are lacking.
- Observational data indicate survival benefits associated with this treatment in children with sepsis, which has led to some PICUs using HAT therapy off-label.
- It is unknown if a protocol of HAT versus standard care is feasible in children with septic shock, and whether this intervention results in shorter survival free of organ dysfunction.

**WHAT THIS STUDY MEANS**

- In this pilot randomized trial including 60 children with septic shock, a protocol comparing high-dose vitamin C, hydrocortisone, and thiamin versus standard care was feasible. The findings of this pilot study can inform the design of a full randomized controlled trial (RCT).
- Rates of parental consent were high, and treatment was delivered at a median of 44 minutes after randomization.
- The assessment of primary outcome—organ dysfunction-free survival—was also a feasible measure, and we estimate a sample size of *n* = 384 for a future RCT.

The highest lifetime incidence of sepsis occurs in childhood, with a worldwide 1990–2017 estimate of annual deaths of 3 million (1, 2). In Australia and New Zealand (ANZ), our most contemporary data, 2006–2016, indicate that about one-in-six children with septic shock admitted to the PICUs die (3). Moreover, survivors frequently suffer serious sequelae, which affect long-term health-related quality of life (4). Most children with septic shock present with rapid deterioration, and in our ANZ 2012–2015 experience, half of the deaths occurred within 48 hours of presentation (5). These observations imply that there is need for early interventions to reverse pediatric septic shock close to the time of presentation.

IV corticosteroids are commonly used to treat pediatric septic shock refractory to initial resuscitation, albeit with no evidence for mortality benefit in children nor adults (6, 7). Recently, adjunctive therapy combining hydrocortisone with high-dose ascorbic acid (vitamin C) and thiamin (HAT therapy, or “metabolic resuscitation”) has been evaluated in adults with sepsis. This therapy is based on preclinical data informed by the antioxidant and anti-inflammatory effects of vitamin C (8, 9), as well as associations of thiamin deficiency with lactatemia (10). HAT therapy has been trailed in adults, but reports have divergent findings on mortality (11–17). There are no similar randomized controlled trials (RCTs) in children with sepsis. A propensity-matched study reported decreased 30-day mortality in 43 children with septic shock treated with vitamin C, hydrocortisone, and thiamin (18). Therefore, we conducted the multicenter Resuscitation in Paediatric Sepsis Using Metabolic Resuscitation—A Randomized Controlled Study in the Paediatric Intensive Care Unit (RESPOND PICU) study to test the hypothesis that a trial of high-dose vitamin C, thiamin, and hydrocortisone would be feasible in children admitted to PICU with septic shock.

## METHODS

### Study Design and Oversight

RESPOND PICU was an investigator-initiated pragmatic, multicenter, randomized, open-label, parallel-group pilot trial endorsed by the ANZ Intensive Care Society (ANZICS) Paediatric Study Group (PSG) (**Supplementary Material S1**, http://links.lww.com/PCC/C421). The trial was registered before the start of recruitment (ACTRN12619000829112). The Child Health Research Centre (The University of Queensland, Brisbane, Australia) managed the trial. The ethics committee approved the protocol (HREC/18/QCHQ/49168, **Supplementary Material S2**, http://links.lww.com/PCC/C421) which was published before completion of enrollment (19). Written informed consent was provided by parents or guardians before enrollment. If timely informed consent was deemed not feasible, the study team was allowed to employ consent to continue until written consent could be obtained. An independent Data and Safety Monitoring Board oversaw the trial (**Supplementary Material S3**, http://links.lww.com/PCC/C421).

### Patients and Settings

Children between 28 days and 18 years old admitted to the PICU were eligible if they received inotropic therapy (at any dose; independent of the site where inotropes were started) for presumed septic shock for greater than or equal to 2 hours, but less than 24 hours. Screening occurred day and night, 7 days a week. We excluded patients with chronic organ dysfunction, those receiving chemotherapy for cancer, and patients who suffered a cardiorespiratory arrest preceding enrollment (**Supplementary Material S4**, http://links.lww.com/PCC/C421). We allowed coenrollment in a study investigating early inotropes in children presenting to the emergency department (ACTRN12619000828123) (20, 21). The trial was initiated at the lead site (Queensland Children`s Hospital, Brisbane) in June 2019. By early 2021, five PICUs in ANZ joined.

### Randomization

Eligible patients were randomized 1:1 to adjunctive therapy with vitamin C, hydrocortisone, and thiamin (intervention), versus septic shock management according to institutional protocols (standard care) (22, 23). Allocation occurred using sealed opaque envelopes which contained the randomization assignment generated by The University of Queensland. Permuted block randomization with variable block sizes of two, four, and six, stratified by site was used. No blinding was performed given the difficulties of blinding three drugs, one of which has a distinct yellow color (12).

### Interventions

Patients allocated to the vitamin C, hydrocortisone, and thiamin group received 30 mg/kg/dose vitamin C (maximum 1,500 mg per dose as sodium ascorbate, Biological therapies) IV every 6 hours, hydrocortisone 1 mg/kg/dose (maximum 50 mg per dose) IV every 6 hours, and thiamin 4 mg/kg/dose (maximum 200 mg per dose) IV every 12 hours immediately after randomization. The dosing schedule has been shown to restore serum ascorbic acid concentrations in septic patients (12, 24). The study treatment was administered for 7 days, until resolution of shock (defined as cessation of inotropes for at least 4 hr), death, discharge from the PICU, or if major adverse events related to the intervention occurred. Patients in the standard treatment arm were allowed to receive hydrocortisone only if the treating PICU staff specialist considered it clinically indicated. Other care in the PICU was provided according to the institutional practice.

### Study Endpoints

The primary feasibility outcomes included recruitment rates, time to initiation of the intervention, proportion of patients receiving hydrocortisone outside the study protocol, and adherence to the study protocol.

The primary clinical efficacy outcome being assessed was defined as survival free of organ dysfunction censored at day 28 after randomization. Organ dysfunction was assessed daily using the pediatric Sequential Organ Failure Assessment (pSOFA [25, 26]) score. Patients who died within 28 days of randomization were considered to have zero organ dysfunction-free days.

Secondary clinical efficacy outcomes being assessed included survival free of inotrope support at 7 days, survival free of multiple organ dysfunction at 7 days, mortality, PICU-free survival, survival free of acute kidney injury (27), and PICU and hospital length of stay. In addition, Pediatric Overall Performance Category (POPC) and Functional Status Score (FSS) were assessed at 28 days using phone or face-to-face interviews and compared with baseline scores (28, 29). Baseline scores referred to “how was your child before this hospital admission” and were obtained with randomization. In addition, we assessed proxy measures of intervention efficacy including shock reversal, normalization of lactate (< 2 mmol/L), and time to reversal of tachycardia (defined by age-specific thresholds for Systemic Inflammatory Response Syndrome [30]).

### Data Collection

The Research Electronic Data Capture database recorded baseline variables, comorbidities, disease and severity features, predefined study outcomes, information on study treatments, and adverse events. Physiological parameters and organ support were collected upon randomization, then at 1, 6, 12, and 24 hours, and daily until discharge from PICU. Data monitoring (**Supplemental Materials S5**, http://links.lww.com/PCC/C421) included primary source data verification in 100% of enrolled patients for randomization allocation, consent, study treatments, organ support, PICU length of stay, survival status, protocol deviations, and adverse events. In addition, in randomly selected 10% of patients other study data were verified using primary source verification.

### Sample Size

An enrollment of 60 of 80 eligible patients would yield a recruitment rate of 75% with a one-sided lower 95% CI limit of 66%, which would meet our key feasibility metric for a recruitment rate set at greater than or equal to 65%. As this is a feasibility trial, no power calculations for the exploratory clinical endpoints were applicable.

### Statistical Analysis

The statistical analysis plan was published, and the full Stata analysis code was uploaded on GitHub before the completion of enrollment (19). Main analyses were performed on the modified intention-to-treat population, which we defined as all enrolled patients except those who were randomized but where parents did not grant consent.

We used descriptive statistics to compare demographics, clinical history, and baseline clinical characteristics between the study arms. For each of the predefined primary and secondary outcomes, the estimate of the difference between the two study groups and the corresponding 95% CI was reported. Quantile regression was used to assess the comparison for continuous outcomes and the test of two proportions for binary outcomes. We performed a predefined sensitivity analysis for primary and secondary outcomes, separating the standard care arm into two groups—those not receiving hydrocortisone, and those receiving hydrocortisone. Given the pilot design, no tests for statistical significance for clinical outcomes were performed. All analyses were performed with Stata/SE, version 17.0 (StataCorp Pty Ltd, College Station, TX).

## RESULTS

### Patients and Recruitment Rates

**Figure 1.**
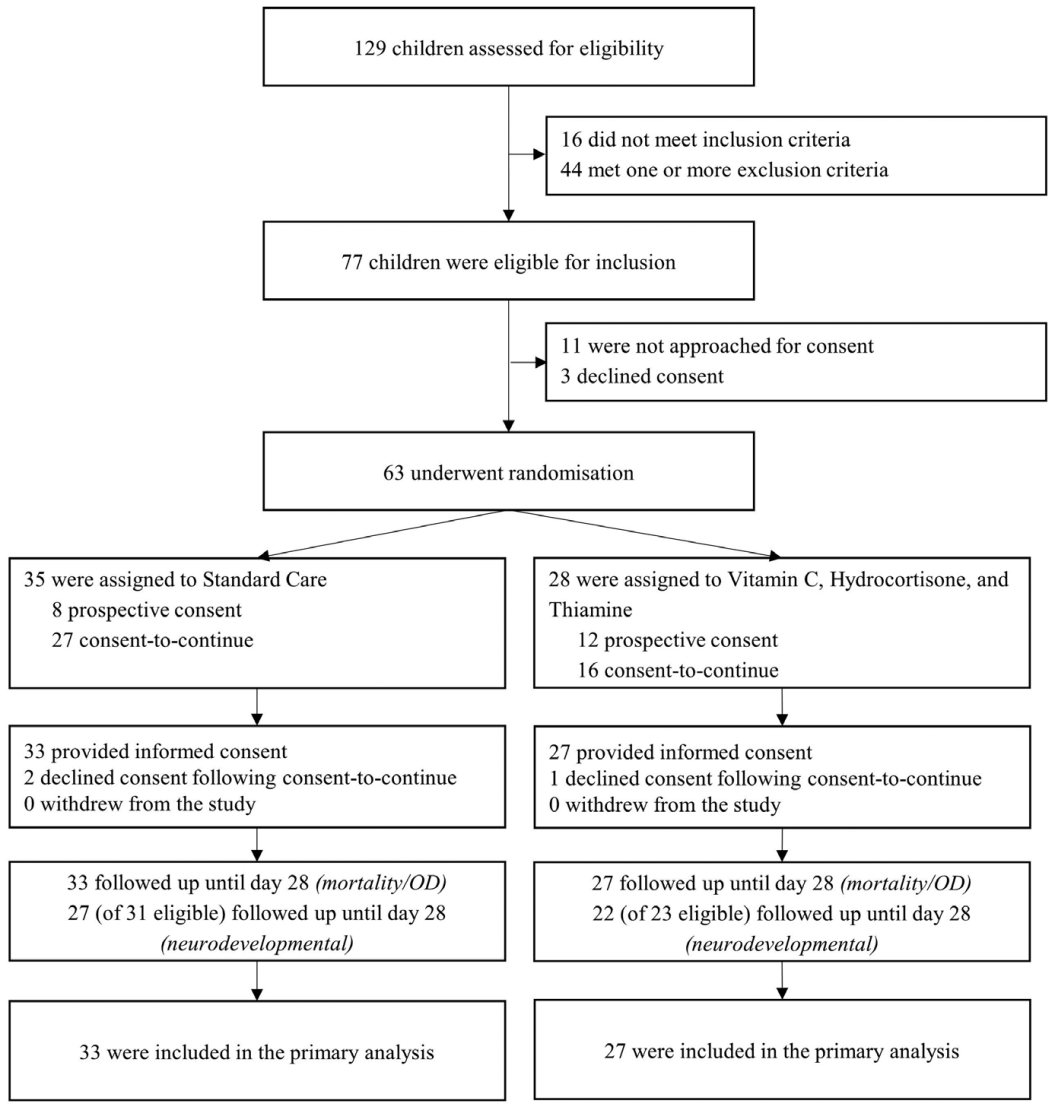
Consolidated Standards of Reporting Trials participant flow diagram for the Resuscitation in Paediatric Sepsis using Metabolic Resuscitation—a Randomized Controlled Pilot Study in the PICU trial.

From August 2019 to March 2021, of 129 screened children, 77 met the eligibility criteria. Of these, 11 of 77 (14%) were not approached for consent, and parents of 3 of 77 (4%) patients declined consent. Thus, we enrolled 63 of 77 (82%) patients, with consent-to-continue used in 43 of 77 (63%). In a further three (4%) randomized patients, parents declined consent following a consent-to-continue approach, leaving a modified intention-to-treat population of 60 of 77 (78%), with 27 assigned to the vitamin C, hydrocortisone, and thiamin intervention, and 33 assigned to standard care (**Fig. 1**; and **Fig. S1**, http://links.lww.com/PCC/C421). The lead site maintained an average recruitment rate of 2.3 patients per month. The median age of enrolled patients was 5.2 (IQR 1.4, 11.8) months, and 27 of 60 (45%) had comorbidities. The baseline characteristics of the study groups are shown in **Table 1**. The median pSOFA score at enrollment was 9 (IQR 7, 11.5) in the intervention group, and 9 (IQR 7, 11) in the standard care group, respectively. Eight of 27 (30%) patients in the intervention group, and 9 of 33 (27%) in the standard group, had received IV steroids before randomization. Forty-nine of 60 (82%) and 47 of 60 (78%) completed POPC and FSS surveys at 28 days, respectively.

**TABLE 1.** Baseline Characteristics of Infants Enrolled in the Resuscitation in Paediatric Sepsis Using Metabolic Resuscitation—A Randomized Controlled Pilot Study in the Paediatric Intensive Care Unit PICU Trial

Characteristic	Standard Care, *n* = 33	Vitamin C, Hydrocortisone, and Thiamin, *n* = 27
Age at randomization (mo), median (IQR)	5.7 (1.5, 11.8)	4.4 (1.1, 13.4)
Weight (kg), median (IQR)	21.0 (11.1, 44.4)	15.6 (10.0, 38.4)
Female sex, *n* (%)	17 (52%)	12 (44%)
Ethnicity, *n* (%)		
Caucasian	10 (30%)	11 (41%)
Aboriginal/Torres Strait Islander	4 (12%)	2 (7%)
Asian	2 (6%)	2 (7%)
Maori/Pacific Islander	3 (9%)	2 (7%)
Mixed/other	3 (9%)	1 (4%)
Unknown	11 (33%)	9 (33%)
Chronic disease^a^, *n* (%)	16 (48%)	11 (41%)
Congenital malformation	1 (3%)	1 (4%)
Congenital heart defect	7 (21%)	3 (11%)
Oncologic disease	1 (3%)	0 (0%)
Cerebral palsy/severe encephalopathy	5 (15%)	4 (15%)
Syndrome/genetic disorder	8 (24%)	3 (11%)
Observations at baseline		
Heart rate, median (IQR)	131 (111, 155)	140 (117, 159)
Respiratory rate (*n* = 55), median (IQR)	28 (25, 33)	27 (22, 32)
Systolic blood pressure, median (IQR)	96 (74, 107)	92 (77, 102)
Temperature (*n* = 51), median (IQR)	37.2 (36.3, 38.4)	38.0 (37.1, 39.1)
Pao_2_/Fio_2_ (*n* = 46) ratio, median (IQR)	270 (158, 364)	228 (176, 337)
Invasive respiratory support, *n* (%)	21 (64%)	20 (74%)
Glasgow Coma Score, median (IQR)	6.0 (3.0, 12.5)	3.0 (3.0, 8.0)
Vasoactive inotrope score, median (IQR)	8.0 (3.0, 20.0)	10.0 (6.0, 23.3)
IV steroids for septic shock, *n* (%)	9 (27%)	8 (30%)
Laboratory, median (IQR)		
pH (*n* = 58)	7.31 (7.25, 7.39)	7.28 (7.20, 7.38)
Base excess (mmol/L) (*n* = 56)	–7.1 (–9.8, –3.4)	–7.1 (–12.6, –0.8)
Lactate (mmol/L) (*n* = 58)	2.1 (1.2, 3.4)	1.7 (1.0, 2.8)
Creatinine (µmol/L) (*n* = 51)	62 (29, 85)	55 (29, 93)
Fibrinogen (g/L) (*n* = 38)	4.2 (2.8, 5.3)	3.1 (2.2, 4.6)
Platelets (×10^3^/µL) (*n* = 49)	146 (69, 225)	178 (107, 218)
WBC Count (×10^3^/µL) (*n* = 51)	11.4 (6.6, 21.5)	11.9 (4.9, 19.2)
Hemoglobin (g/L) (*n* = 51)	105 (92, 118)	116 (98, 128)
C-reactive protein (mg/L) (*n* = 34)	174 (122, 272)	202 (26, 231)
Pediatric Sequential Organ Failure Assessment	9.0 (7.0, 10)	9.0 (7.0, 12.0)

IQR = interquartile range, POPC = Pediatric Overall Performance Category.

a More than one can apply.

### Feasibility of the Protocol

The median time from screening to randomization was 15 (IQR 0, 40) minutes (**Table 2**). Vitamin C, thiamin, and hydrocortisone were administered to 27 of 27 (100%) patients in the intervention group, at a median of 44 (IQR 29, 120) minutes after randomization. In the standard care arm, 0 of 33 (%), 8 of 33 (24%), and 3 of 33 (9%) patients received vitamin C, hydrocortisone, and thiamin, respectively, after randomization. These findings were comparable for the lead site as well as the other study sites (**Tables S1** and **S2**, http://links.lww.com/PCC/C421).

**TABLE 2.** Feasibility Outcomes Per Intention-to-Treat Analysis for All Sites

Outcome	Standard Care, *n* = 33	Vitamin C, Hydrocortisone, and Thiamin, *n* = 27	Estimate of Difference (95% CI)
Time from screening to randomization (min), median (IQR)	15 (0, 40)	30 (8, 90)	15 (–16 to 46)
Time from PICU admission to randomization (min), median (IQR)	340 (117, 815)	372 (170, 1650)	32 (–517 to 581)
Time from randomization to commencement of metabolic resuscitation (min), median (IQR)	Not applicable	44 (29, 120)	Not applicable
Hydrocortisone received after randomization, *n* (%)	8 (24%)	27 (100%)	76% (61% to 90%)
Cumulative hydrocortisone dose after randomization (mg/kg), median (IQR)	13.4 (9.3, 16.9)	5.2 (3.0, 12.0)	–8.0 (–14.9 to –1.1)
Ascorbic acid received after randomization, *n* (%)	0 (0%)	27 (100%)	Not applicable
Cumulative ascorbic acid dose after randomization (mg/kg), median (IQR)	Not applicable	149.3 (90.0, 300.0)	Not applicable
Thiamin received after randomization, *n* (%)	3 (9%)	27 (100%)	91% (81% to 100%)
Cumulative thiamin dose after randomization (mg/kg), median (IQR)	4.0 (2.2, 45.8)	11.9 (4.8, 19.8)	7.9 (–14.4 to 30.1)

IQR = interquartile range.

Overall, 15 protocol violations occurred, affecting 5 of 27 (19%) patients in the intervention group and 7 of 33 (21%) patients in the standard care group (**Table S5**, http://links.lww.com/PCC/C421). Protocol violations affected consent and eligibility (*n* = 5), and study treatments (*n* = 6, of which three related to stopping the study intervention too early or too late).

### Description of Exploratory Clinical Endpoints

**Figure 2.**
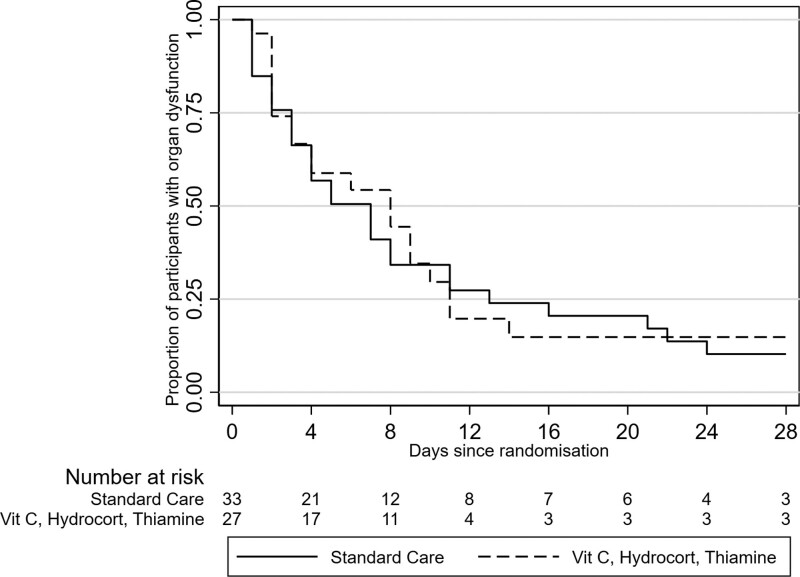
Cumulative incidence functions for survival free of organ dysfunction (accounting for mortality) for the study cohort. Hydrocort = hydrocortisone, Vit C = vitamin C.

At day 28, the median number of organ dysfunction-free days was 20.0 days (IQR 0.0, 26.0) days in the intervention group and 21.0 (IQR 12.0, 25.0) days in the standard care group (unadjusted estimate of absolute difference, –1 days; 95% CI, –10.9–8.9) (**Table 3**, **Fig. 2**). The median survival free of inotrope support at 7 days was 6.3 (IQR 4.1, 7.0) days in the intervention group and 5.9 (4.3, 7.0) days in the standard care group (estimate of difference 0.4; 95% CI, –1.0 to 1.8). Four of 27 (15%) patients in the intervention group and 2 of 33 (6%) in the standard care group died within 28 days after randomization. The median length of stay in PICU was 5.3 (IQR 2.5, 11.3) days in the intervention group and 6.9 (IQR 3.0, 11.5) days in the standard care group (estimate of difference –1.6 d; 95% CI, –6.2 to 3.0). Other secondary outcomes, including comparison of POPC and FSS from baseline to day 28 are provided in Table 3 and Fig. S2 (http://links.lww.com/PCC/C421).

**TABLE 3.** Exploratory Clinical Outcomes Per Intention-to-Treat Analysis

Outcome	Standard Care, *n* = 33	Vitamin C, Hydrocortisone, and Thiamin, *n* = 27	Estimate of Difference (95% CI)
Primary clinical outcome			
Survival free of organ dysfunction^a^ censored at 28 d, median (IQR)	21.0 (12.0, 25.0)	20.0 (0.0, 26.0)	–1 (–10.9 to 8.9)
Secondary clinical outcomes			
Survival free of inotrope support at 7 d, median (IQR)	5.9 (4.3, 7.0)	6.3 (4.1, 7.0)	0.4 (–1.0 to 1.8)
Survival free of multiple organ dysfunction^b^ at 7 d, median (IQR)	2.0 (0.0, 4.0)	2.0 (0.0, 5.0)	0 (–2.4 to 2.3)
Survival free of acute kidney injury at 28 d, median (IQR)	28.0 (27.0, 28.0)	28.0 (28.0, 28.0)	—
28-d mortality, *n* (%)	2 (6%)	4 (15%)	9% (–7% to 24%)
Survival free of PICU censored at 28 d, median (IQR)	20.3 (11.2, 24.5)	20.0 (0.0, 25.5)	–0.3 (–10.2 to 9.5)
Length of stay in PICU, median (IQR)	6.9 (3.0, 11.5)	5.3 (2.5, 11.3)	–1.6 (–6.2 to 3.0)
Length of stay in hospital, median (IQR)	14.7 (8.3, 25.8)	13.6 (6.1, 29.0)	–1.1 (–11.6 to 9.3)
POPC at 28 d (*n* = 49^c^), median (IQR)	3 (2, 5)	3 (2, 4)	0 (–1.4 to 1.4)
Change in POPC from baseline (*n* = 49), median (IQR)	0 (0, 1)	1 (0, 2)	1 (0.2 to 1.8)
Functional Status Score at 28 d (*n* = 47^c^), median (IQR)	8 (6, 13)	9 (7, 13)	1 (–2.9 to 4.9)
Change in Functional Status Score from baseline, median (IQR)	0 (0, 2)	2 (0, 6)	3 (0.9 to 5.1)
Proxy measures of intervention efficacy			
Lactate < 2 mmol/L by 6 hr postenrolment, *n* (%)	24 (73%)	16 (59%)	–13% (–37% to 10%)
Lactate < 2 mmol/L by 12 hr postenrolment, *n* (%)	26 (79%)	19 (70%)	–8% (–31% to 14%)
Lactate < 2 mmol/L by 24 hr postenrolment, *n* (%)	28 (85%)	22 (81%)	–3% (–22% to 16%)
Time to reversal of tachycardia censored at 24 hr, median (IQR)	0 (0, 6.0)	0 (0, 6.0)	0 (–3.1 to 3.1)
Time to shock reversal censored at 28 d (hr), median (IQR)	47.3 (22.4, 106.8)	35.2 (14.6, 101.2)	–12.0 (–56.8 to 32.7)

IQR = interquartile range, POPC = Pediatric Overall Performance Category.

a Measured by pediatric Sequential Organ Failure Assessment (pSOFA) score.

b Multiple organ dysfunction is defined as >1 organ with a pSOFA subscore of > 0.

c Numbers relate to patients where parents could be contacted, responded, and completed the follow-up questionnaire.

### Proxy Measures of Intervention Efficacy

The median time to shock reversal censored at 28 days was 35.2 (IQR 14.6, 101.2) hours in the intervention group and 47.3 (IQR 22.4, 106.8) hours in the standard care group (estimate of difference –12.0 hr; 95% CI, –56.8 to 32.7, see Table 3). Figure S3 (http://links.lww.com/PCC/C421) shows changes in shock index, heart rate, systolic blood pressure, Vasopressor-Inotrope-Score (31), serum lactate levels, and use of fluid bolus volume (in mL/kg) between the intervention group versus controls, starting at the time of randomization.

### Sensitivity Analyses

Preplanned sensitivity analyses were performed on the primary and secondary clinical outcomes comparing the intervention group with patients in the standard care group who had received hydrocortisone (*n* = 8), and those who had not (*n* = 25). Median pSOFA at baseline in standard care patients who received no hydrocortisone was 9 (IQR 6, 9) as compared to 15 (IQR 11, 16) in the standard care group patients receiving hydrocortisone. Median survival free of organ dysfunction censored at 28 days was 20.0 (IQR 0.0, 26.0) days in the intervention group compared to 5.5 (IQR 0.0, 14.5) days in the hydrocortisone standard care group, and 24.0 (IQR 20.0, 26.0) days in the no hydrocortisone standard care group, respectively (Table S3 and Fig. S4, http://links.lww.com/PCC/C421). Additional sensitivity analyses restricted to patients with septic shock and acute lung injury resulted comparably (**Table S4**, http://links.lww.com/PCC/C421).

### Adverse Events

A total of 22 adverse events occurred in 9 of 27 (33%) patients in the intervention group and 8 of 33 (24%) patients in the standard care group (**Table S5**, http://links.lww.com/PCC/C421). One adverse event was related to the intervention: a 15-year-old male was erroneously administered too high a dose of vitamin C (15 g instead of 1.5 g, equaling 242 mg/kg) once. Additional blood tests were performed to check renal function, which was considered an intervention-related adverse event. The patient had no side effects and organ dysfunction resolved within 37 hours of the inadvertent dose (**Figure S5**, http://links.lww.com/PCC/C421).

## DISCUSSION

### Key Findings

In this multicenter open-label randomized pilot trial of children with septic shock, a study protocol delivering IV vitamin C, hydrocortisone, and thiamin demonstrated feasibility. The study achieved enrollment of 78% of eligible patients, with 91% of approached parents or guardians providing written consent. Consent-to-continue was used in two-thirds of enrollments. Given the time-critical nature of the septic shock, we see the rationale to consider consent-to-continue as a preferred approach, but further research into co-designed resources to aid in the consenting process for families and researchers/clinicians is warranted. Screening and randomization occurred at a median of six hours after PICU admission, and the intervention was delivered less than one hour after randomization. Although the study was not powered for clinical endpoints, we did not record any relevant side effects related to the intervention.

### Relationship to Previous Studies

In adults, a major controversy has surrounded high-dose vitamin C, thiamin, and hydrocortisone treatment following conflicting results of recent RCTs. Initial promising reports had led to the adoption of HAT in many international adult and pediatric ICUs. The latest data provide additional controversy to redesign trials on vitamin C in sepsis. First, an ovine sepsis model demonstrated dramatic improvements in cardiac output, blood pressure, and renal perfusion, reduced fever, lower inotrope requirement, and restored urine output and hyperlactatemia after treatment with much higher (>1 g/kg) vitamin C doses than applied in the published RCTs (8). Second, a component network meta-analysis on 32 RCTs including 9,224 adult patients with septic shock identified lower mortality associated with very high doses of vitamin C than with standard care, and potential for harm associated with thiamin which thus should be dropped from a future full RCT (16). Adding glucocorticoid treatment shortened the duration of vasopressor therapy and ICU stay. Third, the recent LOVIT trial observed an increased incidence of the composite outcome of death or persistent organ dysfunction at 28 days in adults with infection requiring vasopressors treated with 50 mg/kg vitamin C compared to placebo (17). More recent meta-analyses indicate a low certainty for early mortality benefit of high-dose vitamin C in adults, but moderate certainty for harm at 90 days (32). Further complexity to this controversy stems from pharmacological formulations, with some trials using sodium ascorbate (as in this trial) and others using ascorbic acid which has a much lower pH.

Until now, only observational data on the combination of vitamin C, hydrocortisone, and thiamin in pediatric age groups has been published (18). Critically ill children with sepsis have very high metabolic turnover and deplete their vitamin C stores rapidly (33, 34). Very-low vitamin C serum levels on admission to the PICU were inversely correlated with multiple organ dysfunction at 24 hours (aOR 3.38; 95% CI, 1.53–7.47) (35). Systematic reviews indicate an excellent safety profile of vitamin C even at doses up to 100 mg/kg/dose and higher (36, 37).

### Implications

Our findings suggest that an RCT of this intervention would be feasible with a high rate of consent and that the intervention could be delivered rapidly. Although this study was not powered for safety outcomes, we did not identify a clear signal towards increased adverse events likely or possibly related to the intervention. The median pSOFA at enrollment was 9 in our study which is comparable to the VITAMINS trial, but even earlier enrollment may be facilitated by predefined Vasoactive Inotrope Score eligibility thresholds. The observed use of hydrocortisone pre-randomization and post-randomization, in particular in the control arm, provides rationale for a three-arm trial design. Although the pragmatic design enabled high recruitment and consent rates in our pilot (38), violations in a full trial as a result of provider preference in the absence of blinding may expose the trial to risks of bias and contribute to dilution or contamination of the study arms. Furthermore, only 82% of parents completed the surveys at 28 days, necessitating targeted education and follow-up strategies.

Based on our data, and powering for a clinically relevant increase in the time alive and free of vasopressors (12) of at least 1 day, we estimate that 384 patients would be needed for a trial comparing vitamin C with hydrocortisone, and hydrocortisone alone, versus controls (128 participants per group) along with 80% power, a corrected type I error of 0.025 to allow for two primary comparisons, attrition of 5% and inflation for a non-normally distributed outcome. We estimate that it would take up to 4 years to recruit for such a powered trial assuming participation of 15–20 PICUs.

### Limitations

Several limitations of this feasibility trial need to be considered. First, due to the difficulties in blinding three drugs, and the characteristic color of vitamin C, treatment concealment was not performed. This leaves risk of bias in outcome ascertainment for the interventions, and frontline clinicians may not have equipoise on vitamin C or hydrocortisone. Second, the study protocol allowed the use of steroids in the control arm, which makes it impossible to assess the confounding of severity on the impact of steroids on outcomes (39). Third, recruitment occurred at ANZICS PSG sites, which does not allow us to define feasibility in low-income and middle-income countries where different baseline risks, pathogens, and comorbidities may introduce further heterogeneity. Fourth, the clinical outcome of organ dysfunction-free adjudicated a zero rather than a negative value to death and did not consider new or progressive multiple organ dysfunction (40).

## CONCLUSIONS

Our findings demonstrate that a pragmatic trial on high-dose vitamin C, hydrocortisone, and thiamin in children with septic shock admitted to PICU is feasible. These findings inform the design of and provide a rationale for a larger trial (ACTRN12621000247875).

## ACKNOWLEDGMENTS

The authors thank the children and families participating in this trial. In addition, we would like to express our gratitude to the PICU staff at the participating sites for their help in the study conduct.

Australian and New Zealand Intensive Care Society Paediatric Study Group (ANZICS PSG): Anusha Ganeshalingam, Claire Sherring, Starship Children’s Hospital, Auckland, New Zealand; Simon Erickson, Samantha Barr, Perth Children`s Hospital, Perth, Australia; Sainath Raman, Debbie Long, Luregn Schlapbach (Past Chair), Kristen Gibbons (Vice Chair), Queensland Children’s Hospital and The University of Queensland, Brisbane, Australia; Shane George, Gold Coast University Hospital; Puneet Singh, Vicky Smith, Sydney Children’s Hospital, Randwick, Australia; Warwick Butt (Chair), Carmel Delzoppo, Johnny Millar (ANZPIC Registry Lead), Royal Children’s Hospital, Melbourne, Australia; Breanna Pellegrini (ANZPIC Registry); Felix Oberender, Monash Children`s Hospital, Melbourne, Australia; Subodh Ganu, Georgia Letton, Women’s and Children’s Hospital, Adelaide, Australia; Jennifer Darvas, Marino Festa, Children’s Hospital Westmead, Sydney, Australia.

## Supplementary Material


