# Supplementary material for: Resuscitation With Vitamin C, Hydrocortisone, and Thiamin in Children With Septic Shock: A Multicenter Randomized Pilot Study
Source: Pediatr Crit Care Med. 2024 Jan 19;25(2):159–70. doi: 10.1097/PCC.0000000000003346 (PMC10793796; doi:10.1097/PCC.0000000000003346)

***Supplementary Materials to***

**Resuscitation with Vitamin C, Hydrocortisone, and Thiamine in Children with Septic Shock - a Multicenter Randomized Pilot Study**

**The RESPOND PICU Randomized Clinical Trial**

Luregn J Schlapbach^1,2,3*^, MD, PhD, FCICM, Sainath Raman^1,2^, PhD, FCICM, David Buckley^4^, MBChB, FCICM, Shane George^1,5,6^, FACEM, Megan King^5^, FACEM, Roberta Ridolfi^1,2^, GradCert, Amanda Harley^1,5,7^, PhD, Michele Cree^1,2^, MBBS, Debbie Long^1,8^, PhD, Simon Erickson^9^, MBBS, FCICM, Puneet Singh^10^, FCICM, Marino Festa^11,12^, MD (Res), FCICM, Kristen Gibbons^1^, PhD, Rinaldo Bellomo^13,14,15,16^, PhD, FCICM, on behalf of the RESPOND PICU study investigators and the Australian and New Zealand Intensive Care Society Paediatric Study Group (ANZICS PSG)

*corresponding author

^1^ Child Health Research Centre, The University of Queensland, Brisbane, Queensland, Australia

^2^ Paediatric Intensive Care Unit, Queensland Children's Hospital, Children's Health Queensland, Brisbane, Queensland, Australia

^3^ Department of Intensive Care and Neonatology, and Children's Research Center, University Children's Hospital Zurich, University of Zurich, Zurich, Switzerland

^4^ Paediatric Intensive Care Unit, Starship Children’s Hospital, Auckland, New Zealand

^5^ Departments of Emergency Medicine and Children’s Critical Care, Gold Coast University Hospital, Southport, Australia

^6^ School of Medicine and Menzies Health Institute Queensland, Griffith University, Southport, Australia

^7^ School of Nursing, Midwifery and Social Work, University of Queensland, QLD Australia

^8^ School of Nursing, Centre for Healthcare Transformation, Queensland University of Technology, Brisbane, QLD, Australia

^9^ Paediatric Critical Care Unit, Perth Children`s Hospital, Perth, Australia

^10^ Paediatric Intensive Care Unit, Sydney Children’s Hospital, Sydney, Australia

^11^ Paediatric Intensive Care Unit, Children’s Hospital Westmead, Sydney, Australia

^12^ Kids Critical Care Research Group, Kids Research, Sydney Children’s Hospitals Network, Sydney, Australia

^13^ Intensive Care Research, Austin Hospital and Monash University, Melbourne, Australia

^14^Department of Critical Care, University of Melbourne., Melbourne, Australia

^15^Australian and New Zealand Research Centre, Monash University, Melbourne, Australia

^16^Department of Intensive Care, Royal Melbourne Hospital, Melbourne, Australia

**Corresponding author:**

Prof. Luregn Schlapbach, MD, PhD

Head, Department of Intensive Care and Neonatology

University Children's Hospital Zurich – Eleonore Foundation

Steinwiesstrasse 75

CH-8032 Zurich Switzerland

phone +41 44 266 71 11

email: l.schlapbach@uq.edu.au

**List of Supplementary Materials**

**Methods**

**S1. RESPOND PICU Study Group**

**S2. Ethics approvals**

**S3. Data and Safety Monitoring Board Charter**

**S4. Inclusion and exclusion criteria**

**S5. Data monitoring plan**

**S6. Data sharing statement**

**Results**

**Figure S1. CONSORT participant flow diagram for the RESPOND PICU trial.**

**Table S1. Feasibility outcomes per intention-to-treat-analysis restricted to the lead study site.**

**Table S2. Feasibility outcomes per intention-to-treat-analysis excluding the lead study site.**

**Table S3. Primary and secondary clinical outcomes per sensitivity analysis comparing children with the intervention (vitamin C, thiamine, hydrocortisone), with children in the standard arm treated with hydrocortisone alone, and without hydrocortisone.**

**Table S4. Primary and secondary clinical outcomes per sensitivity analysis for subgroup of patients with septic shock and acute lung injury.**

**Figure S2. Pediatric Overall Performance Category (POPC) and Functional Status Score (FSS) are compared beween baseline (at randomization) and 28-days after randomization for patients in the standard care, versus patients in the intervention group.**

**Figure S3.** **Composite figure of a) pSOFA, b) heart rate, c) systolic blood pressure, d) VIS score, e) lactate levels, and f) fluid bolus volume administered (in ml/kg) shown at 0, 6, 12, and 24 hours after randomisation, separated by treatment group.**

**Figure S4.** **Cumulative incidence functions for survival free of organ dysfunction (accounting for mortality) per sensitivity analysis comparing children with the intervention (vitamin C, thiamine, hydrocortisone), with children in the standard arm treated with hydrocortisone alone, and without hydrocortisone.**

**Table S5. Protocol violations and major adverse events.**

**Figure S5. Clinical course in a study patient receiving a dose of 15 g total vitamin C.**

**S1. RESPOND PICU Study Group**

Queensland Children`s Hospital: A/Prof Luregn Schlapbach, Dr Sainath Raman, Ms Amanda Harley, Ms Roberta Ridolfi, Ms Natalie Sharp, Ms Michele Cree, A/Prof Debbie Long, Ms Zoe Sever, Ms Kerry Johnson

Austin Hospital and Monash University, Melbourne: Prof. Rinaldo Bellomo

Gold Coast University Hospital: Dr Megan King, A/Prof. Shane George, Mr Nathan Goddard, Mr Kieran Owen

Perth Children’s Hospital, Perth: Dr Simon Erickson, Ms Hannah Thomson

Sydney Children’s Hospital, Sydney: Dr Puneet Singh, Ms Vicki Smith

The Children’s Hospital at Westmead, Sydney: Dr Marino Festa, Dr Chong Tien Goh, Ms Gale Harper

Starship Children’s Hospital, Auckland, NZ: Dr David Buckely, Dr John Beca, Ms Claire Sherring

The University of Queensland: A/Prof Kristen Gibbons, Ms Renate Le Marsney, Ms Trang Pham, Mr Endrias Ergetu

**Australian and New Zealand Intensive Care Society Paediatric Study Group (ANZICS PSG):**

Anusha Ganeshalingam, Claire Sherring, Starship Children’s Hospital, Auckland, New Zealand; Mairead Heaney, Perth Children`s Hospital, Perth, Australia; Sainath Raman, Debbie Long, Luregn Schlapbach (Past Chair), Kristen Gibbons (Vice-Chair), Queensland Children’s Hospital and The University of Queensland, Brisbane, Australia; Shane George, Gold Coast University Hospital; Puneet Singh, Vicky Smith, Sydney Children’s Hospital, Randwick, Australia; Warwick Butt (Chair), Carmel Delzoppo, Johnny Millar (ANZPIC Registry Lead), Royal Children’s Hospital, Melbourne, Australia; Breanna Pellegrini (ANZPIC Registry); Felix Oberender, Monash Children’s Hospital, Melbourne, Australia; Subodh Ganu, Georgia Letton, Women’s and Children’s Hospital, Adelaide, Australia; Jennifer Darvas, Marino Festa, Children’s Hospital Westmead, Sydney, Australia.

**S2. List of ethics approval numbers and approved protocol modifications.**

*Children’s Health Queensland Human Research Ethics Committee approval number:*

**HREC/18/QCHQ/49168** *(Australia), approved 18/12/2018*

| **Revision Chronology:**  The below table provides the detailed list of changes to the protocol. No major changes relating to the PICO (patient, intervention, control, outcomes) were added after the recruitment of the first patient (August 2019). In addition to formal updates relating to study sites/investigators, the changes relate to improving clarity on study analyses, and adding specifics to the operationalization of outcomes and eligibility criteria.   \| **Date of change** \| **Summary of changes** \| \| --- \| --- \| \| **09/04/2019 (prior to commencement of screening)** \| - Study acronym RESPOND added - New study sites and investigators added - Additional detail provided on aspects which are different between the ED and the PICU intervention, including inclusion/exclusion criteria, algorithm and analysis plan - Study algorithm added - Adaptation of study protocol to be feasible at sites which are not using the Queensland Sepsis Pathway - Further details on drug intervention added as per recommendation from QCH Critical Care pharmacist; Thiamine dose adapted to 4mg/kg/dose based on their recommendation - Specified that bloods from this study will be used for the RAPIDS study - More detail on planned follow-up, including shifting to 6 months post-recruitment, and tools used - Specification on consent process regarding consent for 2 interventions and biobanking \| \| **13/06/2019** \| - Additional detail provided on the functional assessment of patients at baseline (i.e. during initial hospitalisation using Functional Status Score), and at 6 month follow-up, and aligned consent form updates \| \| **14/10/2019** \| - New study sites and investigators added - ANZICS Clinical Trial Group endorsement added - Additional funding bodies added - Shortening of Background text, in particular Introduction - Commencement and completion date updated - Clarity added that the two interventions are performed and analysed as related but separate studies, while maximizing the benefit and synergisms of joint conduct with some overlap - Specification of the definition of organ dysfunction used to operationalize the primary outcome (pSOFA score). - Improved clarity of inclusion/exclusion criteria:   - Clarity around including children who are 28 days of age   - Active anti-cancer chemotherapy added as exclusion criterion for the metabolic study and children on inotrope infusion for early inotrope study.   - “Enrolment in RESPOND study <6 months ago (except for RESPOND ED randomization prior to RESPOND PICU within the same sepsis episode)” added as exclusion criteria for either RESPOND ED and PICU   - Clarification added that the early inotrope study (RESPOND ED) will allow recruitment in PICU as well (inclusive of clarification within consent forms) - Flow-charts updated and simplified - Randomisation procedure specified - Aspects of non-protocolised local care specified (pragmatic design) - Clarification of parental questionnaires to be performed at 28 days and 6 months - Additional detail on statistical analyses \| \| **29/03/2020** \| - New study sites and investigators added/updated - Antibiotic duration and consumer satisfaction removed from balancing measures - Update on infusion time for Thiamine and Vitamin C, after discussion with Pharmacy Lead - Stipulation of Renal Replacement Therapy requirement for definition of Chronic Kidney Disease - Further detail provided on follow-up assessment \| \| **17/04/2020** \| - Submission of additional investigators CVs: Gannon, Ridolfi, Cree, Sharp, Raman, Mills, Funk, Tien. Removal of sites. \| \| **22/07/2020** \| - Updated study sites and investigators - Amanda Harley added as main HREC contact - Sainath Raman added as QCH PICU main site investigator - Statement regarding consenting during COVID-19 - Volume of blood reduced per sampling point (no citrate tube) - 2nd blood sampling time point added if feasible \| |
| --- | --- | --- | --- | --- | --- | --- | --- | --- | --- | --- | --- | --- | --- | --- |

**S3. Data and Safety Monitoring Board Charter.**

An independent Data and Safety Monitoring Board (DSMB) has been convened to assess the progress of the RESuscitation in Paediatric Sepsis – a RandOmized coNtrolled (RESPOND) clinical study as well as the safety data and provide recommendations to RESPOND study team. The members of the DSMB serve in an individual capacity and provide their expertise and recommendations. The DSMB will review cumulative study data to evaluate safety, study conduct, and scientific validity and data integrity of the study. This Charter will outline the roles and responsibilities and serve as the Standard Operating Procedure (SOP) for the DSMB.

# COMPOSITION OF THE DSMB

The Board will be composed of four members (inclusive of the DSMB Chair). The DSMB includes experts in, or representatives of, the fields of pediatric intensive care medicine, emergency medicine, and statistics.

Quorum – A quorum will occur when one statistician and one clinician, including the Chair (unless otherwise agreed), occurs. Without a quorum a meeting will not be held, unless alternate arrangements have been made by the Chair in agreement with RESPOND Study Management Team, where documents can be reviewed remotely and written review comments will be provided to the Chair. A separate electronic or documented review of materials may also be requested of members in certain circumstances.

Each member will be appointed for the length of the RESPOND Pilot study.

# INDEPENDENCE OF THE DSMB

It is essential that the judgment of members of the DSMB not be influenced by factors other than those necessary to maintain subject safety and to preserve the integrity of the study. Persons who have an apparent financial, intellectual, or other interests the device, or procedure should not be a DSMB participant for the evaluation of that product. Independence is essential to ensure that DSMB members are objective and capable of an unbiased assessment of the study's safety and efficacy data. The following will ensure the independence of the DSMB:

- DSMB members will not be supervised by any study investigator of a protocol currently under review by this DSMB, or participate as investigators in any study currently under review by this DSMB.
- Members of the DSMB must not have a direct interest in knowing or influencing trial outcome or have a financial or intellectual interest in the outcome of any studies under review.
- DSMB members must disclose all pharmaceutical companies, biotechnology companies, and CROs in which they hold financial interest. Members must disclose all consultancies (direct or indirect) with pharmaceutical companies, biotechnology companies, and CROs.
- Members who have served initially on protocol review teams may participate in the open sessions of the DSMB meeting when that protocol is under review. However, they will be excused from the closed sessions reviewing that protocol.

The RESPOND Coordinating Principal Investigator will be responsible for deciding whether consultancies or the disclosed interests of the members materially affect their objectivity. Members of the DSMB will be responsible for notifying the DSMB Chair and RESPOND Study Management Team of any changes of interest in pharmaceutical companies, biotechnology companies, or CROs, including consultancies. In such cases, the DSMB meeting minutes will document the disclosure of the potential conflict of interest and the outcome of the discussion (e.g., abstention of member from voting, recusal from discussion). The RESPOND Coordinating Principal Investigator will decide whether any of these relationships results in a conflict of interest which would preclude involvement on the DSMB. Members of the DSMB who develop potential or significant perceived conflicts of interest will be asked to resign from the DSMB. Members will be polled at the beginning of each DSMB meeting to disclose whether status has changed.

# RESPONSIBILITIES OF THE DSMB

As this DSMB is constituted for a single protocol, DSMB members should only agree to serve if they are generally supportive of the study’s overall aims and general design. This is because the study has already been through a scientific review. The DSMB will consider study-specific data as well as current relevant background knowledge about the disease, test agent, equipment or patient population under study.

## Objectives

The primary objective of the DSMB is to monitor the safety of the intervention and the validity and integrity of the data from the RESPOND trial. Additionally, the DSMB will evaluate the pace of recruitment and will make recommendations to the RESPOND Study Management Team regarding the continuation, modification, or termination of any or all arms of the study.

## General Responsibilities

The general responsibilities of the DSMB are:

- To evaluate, on an ongoing basis, the accumulating safety assessments to ensure the ongoing safety of study subjects
- To consider factors external to the study when relevant information becomes available, such as scientific or therapeutic developments that may have an impact on the safety of the participants or the ethics of the study
- To review all documents upon notification to the DSMB
- To review the conduct of the study, including protocol violations
- To review data on participant recruitment, accrual, and retention, as well as assessments of data quality, completeness, and timeliness
- DSMB members will have the ability to review unmasked clinical data. Unmasked clinical data will be discussed only during the closed session of the meeting when only DSMB members are present.
- Protect the confidentiality of the study data and the DSMB discussions
- To make recommendations to continue, modify, or terminate the study

# DSMB CHAIR RESPONSIBILITIES

The following responsibilities are those of the DSMB Chair:

- Serves as a voting member
- Facilitates the meetings, assists in the development of the agenda, and ensures that the meeting minutes and recommendation(s) are appropriately documented
- Serves as the primary contact person for the DSMB
- Reviews and approves the Charter
- Ensures that those involved in the day-to-day management of the study are excluded from DSMB voting procedures
- Discusses DSMB recommendations with RESPOND Study Management Team and other appropriate members of the project team via teleconference. This responsibility may be delegated to the Co-chair.
- Takes and maintains minutes from closed sessions of DSMB teleconferences until study termination when the minutes are transferred to the custody of RESPOND Study Management Team

# MEETINGS OF THE DSMB

## Organisational Meeting

The first meeting of the DSMB will be an organisational meeting. This meeting will formally establish the DSMB and begin to acquaint the DSMB members with the protocol or types of protocols that this DSMB will be charged with monitoring. It affords the DSMB an opportunity to recommend final revisions to the Charter and the communication plan between the DSMB, the RESPOND Study Management Team.

## Scheduled Protocol and Data Review Meetings

Each protocol and data review meeting will consist of three sessions: Open Session, Closed Session, and Closed Executive Session.

## Open Session

This will begin with an introductory session that includes introductions, roll call, assurance of a quorum, a reminder about the confidential nature of the proceedings and corresponding documentation, and a review of conflict of interest for all DSMB members.

Following the introductory session, the DSMB will move into the open session. Attendees will include the DSMB members, voting and *ex officio* members, the lead study investigator(s) and other study staff personnel, and appropriate Steering Board members. This session may also be open to others as appropriate and upon invitation.

The open session will serve as a general study update. The RESPOND Coordinating Principal Investigator will be called upon to present study status and known relevant findings. Others with specific safety experience or concerns may also be called upon to present. The session will provide a forum for an exchange of information among the various groups involved in the conduct of the study. It will afford the DSMB members an opportunity to question the project team about the study and to seek additional information deemed relevant to the data review. Discussions may include progress of the study, including adverse events, disease status of participants, comparability of groups with respect to baseline factors, protocol compliance, site performance, quality control, and timeliness and completeness of follow-up. Only masked data will be reviewed and/or discussed during the open session.

## Closed Session

Following the open session of the meeting, a closed session involving the DSMB members will be held to review unmasked and/or grouped safety data, discuss findings, and develop and vote on recommendations. During this session, any issues related to subject safety will be discussed. Requests by DSMB Members for the unmasking of data may be made at this time or prior to the meeting. An unmasked statistician should be available to provide guidance and answer questions.

## Closed Executive Session

A brief teleconference will be held between the DSMB Chair and the specified RESPOND Study Management Team to discuss the recommendations of the DSMB.

## Unscheduled Meetings/Reports

Unscheduled meetings can be requested by any party with the responsibility of overseeing the study.

# COMMUNICATION

## Reports to the DSMB

Associated SAEs and AEs, will be provided to the DSMB yearly or as requested by the Board.

Study status reports will be provided to the DSMB at least two weeks prior to each scheduled meeting.

## DSMB Minutes

The DSMB meetings may be audio recorded for the purpose of documenting meeting minutes. Once the Chair approves the minutes, the recordings will be destroyed.

The RESPOND Study Management Team will prepare the draft meeting minutes of the open session and forward to the DSMB Chair, RESPOND Study Management Team for review within one (1) week following the DSMB meeting. Minutes of the open session will describe the proceedings. Draft minutes will be distributed to named attendees for review and comment.

Minutes of the closed session will describe the proceedings of the closed session. Minutes will be taken by the DSMB Chair. If unmasked information is reviewed during the closed session, minutes containing unmasked information will be marked as “Confidential” and distributed to the members of the DSMB only.

At the conclusion of the study, a complete set of the minutes of the closed sessions and the closed reports will be sent to RESPOND Study Management Team.

## Recommendations

Following the closed session, a brief teleconference will be held between the DSMB Chair, RESPOND Study Management Team to discuss the recommendations of the DSMB.

The DSMB can recommend to RESPOND Study Management Team that the current study continue without modification, continue with specified modifications, discontinue one or more arms of the study, or halt or modify the study until more information is available.

# REFERENCES

Department of Health, Education, and Welfare, Office of the Secretary, Protection of Human Subjects. Belmont Report: Ethical Principles and Guidelines for the Protection of Human Subjects of Research, Report of the National Committee for the Protection of Human Subjects of Biomedical and Behavioral Research. DHEW Publication No. (OS) 78-0013 and No. (OS) 78-0014. 18 April 1979.

Ellenberg, Susan S., Fleming, Thomas R., DeMets, David L. Data Monitoring Committees in Clinical Trials, A Practical Perspective. John Wiley and Sons, LTD, West Sussex, England, 2002.

The Greenberg Report (1988). Organization, review, and administration of cooperative studies: a report from the heart special project committee to the National Advisory Heart Council. Controlled Clinical Trials 9 (2): 137-148.

US Food and Drug Administration (2006) Guidance for Clinical Trial Sponsors on the Establishment and Operation of Clinical Trial Data Monitoring Committees. Rockville, MD: FDA. <http://www.fda.gov/RegulatoryInformation/Guidances/ucm127069.htm>.

UNICEF/UNDP/World Bank/WHO (31 March 2004). Operational Guidelines for the Establishment and Functioning of Data and Safety Monitoring Boards. Geneva, Switzerland.

National Institute of Health (May 1, 2001). Policy and Guidelines for Data and Safety Monitoring.

DAMOCLES Study Group (2005). A proposed charter for clinical trial data monitoring committees; helping them do their job well. Lancet 365:711-22.

**S4. Inclusion and exclusion criteria.**

| **Rule** | **Criterium** | **Definition** |
| --- | --- | --- |
| ***Inclusion*** | *Age* | Aged ≥28 days and <18 years |
|  | *Illness* | Admitted to PICU and treated for septic shock |
|  | *Treatment* | Received inotropes for at least 2 hours |
|  | *Consent* | Parental/caregiver consent prior to or after enrolment |
| ***Exclusion*** | *Age* | Preterm babies born <34 weeks gestation that have a corrected age of <28 days |
|  | *Treatment* | Received inotropes for >24 hours pre-enrolment  Patient is receiving treatment for systemic fungal infection or has documented strongyloides infection at the time of randomization  Patient undergoing active chemotherapy for cancer treatment |
|  | *Co-morbidities* | Cardiomyopathy (not due to sepsis) or chronic cardiac failure  Chronic hypertension due to cardiovascular or renal disease, requiring regular antihypertensive treatment.  Known chronic renal failure (defined as requiring Renal Replacement Therapy)  Known chronic hepatic failure (defined as pediatric Sequential Organ Failure Assessment hepatic subscore >0)  Known diseases affecting the steroid axis, including pituitary disease, congenital adrenal hypoplasia, Cushing or Addison's disease  Known glucose-6 phosphate dehydrogenase (G-6PD) deficiency  Patients with known history of oxalate nephropathy  Patients with acute beri-beri disease  Patients with acute Wernike's encephalopathy  Patients with known malaria  Patients with known of suspected scurvy  Palliative care patient/patient with limitation of treatment (not for inotropes, cardiopulmonary resuscitation, extracorporeal membrane oxygenation, intubation or ventilation) |
|  | *Illness severity* | Cardiopulmonary arrest in the past two hours requiring cardiopulmonary resuscitation of >2 minutes duration, or death is deemed to be imminent or inevitable during this admission.  Major bleeding with haemorrhagic shock  Sepsis is not likely to be the cause of shock |
|  | *Previous study enrolment* | Enrolment in RESPOND study <6 months prior |

**S5. Data monitoring plan.**

| **Version No.** | **Version Date** | **Major Changes** |
| --- | --- | --- |
| Version 1.0 | 21/07/2020 | Initial version |

**Purpose**

This data monitoring plan describes the nature and extent of data monitoring activities to be performed for RESuscitation in Paediatric Sepsis – a RandOmized coNtrolled (RESPOND) trial. It was developed by the Coordinating Principal Investigator in collaboration with the study management team, after the conduct of a risk assessment.

Monitoring activities will be performed in accordance with:

1. The Integrated Addendum to ICH E6 (R1): Guideline for Good Clinical Practice E6 (R2) *(ICH-GCP)*;
2. Risk-based management and Monitoring of Clinical Trials Involving Therapeutic Goods, NHMRC, 2018;
3. Protocol-specific requirements; and
4. Applicable policies and procedures at each participating site.

**Roles and Responsibilities**

The data monitoring plan for this study will be developed and implemented by investigators from the study site, the Queensland Children’s Hospital (QCH), along with investigators from The University of Queensland. The site data will be monitored by staff from QCH not involved in the conduct of the trial.

All Monitors are qualified by education and experience to monitor the conduct of the study according to applicable standard operating procedures (SOPs), ICH-GCP, and local requirements.

**Risk Assessment**

Prior to the development of the data monitoring plan, critical data and processes were identified by the Coordinating Principal Investigator in collaboration with the study management team. A risk assessment was conducted to determine the extent and nature of data monitoring activities proportionate to the risk associated with the critical data and processes. The risk assessment was conducted in accordance with the research group’s Data Monitoring Work Instruction and Risk Assessment Template.

**Overview of Monitoring Activities**

There will be three types of data monitoring used for this study:

1. Onsite monitoring or remote monitoring will be conducted routinely to verify that data recorded on the case report forms are accurate, complete, and verifiable from source documentation. Source data verification of all screening eligibility data items, randomisation stratification data items, data items relating to the calculation of primary and secondary outcomes and protocol deviation and adverse event data items will be completed for every enrolled patient. Source data verification on key data items relating to cohort descriptors, as well as source data review to ensure reporting of all relevant protocol deviations and adverse events will be completed for a random sample of 10% of enrolled patients. The original REDCap study database will be enhanced to facilitate the source data verification of the relevant data items.
2. Remote monitoring by the coordinating site will be conducted routinely to review completeness and accuracy of consent forms uploaded to the REDCap study database, and to review automated range and logic discrepancies generated from data quality rules in the REDCap study database.
3. Centralised monitoring will be conducted routinely to evaluate rates of recruitment and withdrawals across study sites.

**Monitoring Reports**

Source data verification results will be documented in a data monitoring report. This report will be generated from the data verified by the monitor in the verification forms in the REDCap study database.

The study site will complete any required updates to the case report form data in the REDCap study database in a timely manner.

**Review**

The Coordinating Principal Investigator in collaboration with the study management team will review the data monitoring plan in response to outcomes of monitoring activities that identify deficiencies and new risks that were not previously considered. Where applicable, an updated version of the monitoring plan will be issued.**S6. Data sharing statement.**

Data available: Yes

Data types: Other

Data types: Deidentified participant data

How to access data: Deidentified participant data will be shared upon reasonable request, addressed to the Corresponding Author at l.schlapbach@uq.edu.au. When available: by 31/12/2022 if published, otherwise at time of publication.

Who can access the data: Researchers with an approved proposed use, approved by appropriate institutional review boards and the Trial Steering Committee.

Types of analyses: An approved specified purpose.

Mechanisms of data availability: With a signed data access agreement.
How to access data: Deidentified participant data will be shared upon reasonable request, addressed to the Corresponding Author at l.schlapbach@uq.edu.au.

When available: With publication

Document types: Statistical/analytic code

How to access documents: The statistical analysis plan has been published before completion of recruitment (Schlapbach LJ, Gibbons K, Ridolfi R, et al. Resuscitation in Paediatric Sepsis Using Metabolic Resuscitation–A Randomized Controlled Pilot Study in the Paediatric Intensive Care Unit (RESPOND PICU): Study Protocol and Analysis Plan. *Frontiers in Pediatrics*. 2021;9doi:10.3389/fped.2021.663435). This publication provides the link to the GitHub repository where the full Stata code was uploaded before completion of recruitment: https://github.com/kgibbons44/RESPONDPilotAnalysis

When available: With publication

Who can access the data: Researchers with an approved proposed use, approved by appropriate institutional review boards and the Trial Steering Committee.

Types of analyses: An approved specified purpose.

Mechanisms of data availability: With a signed data access agreement.

Any additional restrictions: Any data release needs to be approved by the RESPOND PICU Study Group, and the ANZICS Paediatric Study Group (PSG).

**Table S1. Feasibility outcomes per intention-to-treat-analysis restricted to the lead study site (Queensland Children’s Hospital, Brisbane, Australia).**

| **Outcome** | **Standard Care**  **N=20** | **Vitamin C, Hydrocortisone, and Thiamine**  **N=19** | **Estimate of Difference (95% CI)** |
| --- | --- | --- | --- |
| Time from screening to randomisation [min] *median (IQR)* | 16 (0, 78) | 40 (8, 255) | 24 (-75, 123) |
| Time from PICU admission to randomisation [min] *median (IQR)* | 412 (160, 1056) | 201 (81, 681) | -229 (-703, 245) |
| Time from randomisation to commencement of metabolic resuscitation [min] *median (IQR)* |  | 34 (15, 114) |  |
| Hydrocortisone received after randomisation *n (%)* | 4 (20%) | 19 (100%) | 80% (62%, 98%) |
| Cumulative hydrocortisone dose after randomisation [mg/kg] *median IQR* | 11.6 (9.3, 14.1) | 4.8 (3.0, 13.0) | -8.4 (-20.4, 3.6) |
| Ascorbic acid received after randomisation *n (%)* | 0 (0%) | 19 (100%) | - |
| Cumulative ascorbic acid dose after randomisation [mg/kg] *median IQR* | - | 145.2 (90.0, 330.0) | - |
| Thiamine received after randomisation *n (%)* | 2 (10%) | 19 (100%) | 90% (77%, 100%) |
| Cumulative thiamine dose after randomisation [mg/kg] *median IQR* | 3.1 (2.2, 4.0) | 11.9 (7.9, 24.0) | 9.6 (-10.6, 29.8) |

CI, confidence interval; IQR, interquartile range; PICU, paediatric intensive care unit

**Table S2. Feasibility outcomes per intention-to-treat-analysis excluding the lead study site.**

| **Outcome** | **Standard Care**  **N=13** | **Vitamin C, Hydrocortisone, and Thiamine**  **N=8** | **Estimate of Difference (95% CI)** |
| --- | --- | --- | --- |
| Time from screening to randomisation [min] *median (IQR)* | 14 (0, 30) | 25 (6, 45) | 16 (-18, 50) |
| Time from PICU admission to randomisation [min] *median (IQR)* | 269 (111, 375) | 1273 (565, 2865) | 1381 (286, 2476) |
| Time from randomisation to commencement of metabolic resuscitation [min] *median (IQR)* |  | 124 (65, 156) |  |
| Hydrocortisone received after randomisation *n (%)* | 4 (31%) | 8 (100%) | 69% (44%, 94%) |
| Cumulative hydrocortisone dose after randomisation [mg/kg] *median IQR* | 16.1 (9.1, 19.4) | 6.1 (3.6, 8.8) | -6.8 (-16.7, 3.3) |
| Ascorbic acid received after randomisation *n (%)* | 0 (0%) | 8 (100%) | - |
| Cumulative ascorbic acid dose after randomisation [mg/kg] *median IQR* | - | 156.7 (66.2, 217.2) | - |
| Thiamine received after randomisation *n (%)* | 1 (8%) | 8 (100%) | 92% (78%, 100%) |
| Cumulative thiamine dose after randomisation [mg/kg] *median IQR* | 45.8 (-) | 11.5 (4.4, 15.8) | - |

CI, confidence interval; IQR, interquartile range; PICU, paediatric intensive care unit

**Table S3. Primary and secondary clinical outcomes per sensitivity analysis comparing children with the intervention (vitamin C, thiamine, hydrocortisone), with children in the standard arm treated with hydrocortisone alone, and without hydrocortisone.**

| **Outcome** | **Standard Care without Hydrocortisone**  **N=25** | **Standard Care with Hydrocortisone**  **N=8** | **Vitamin C, Hydrocortisone, and Thiamine**  **N=27** |
| --- | --- | --- | --- |
| **Primary clinical outcome** |  |  |  |
| Survival free of organ dysfunction^*^ censored at 28 days *median (IQR)* | 24.0 (20.0, 26.0) | 5.5 (0.0, 14.5) | 20.0 (0.0, 26.0) |
| **Secondary clinical outcomes** |  |  |  |
| Survival free of inotropic support at 7 days *n (%)* | 6.1 (5.2, 7.0) | 3.1 (0.9, 6.3) | 6.3 (4.1, 7.0) |
| Survival free of multiorgan dysfunction^**^ at 7 days *n (%)* | 3.0 (0.0, 5.0) | 0.0 (0.0, 0.5) | 2.0 (0.0, 5.0) |
| Survival free of AKI at 28 days *median (IQR)* | 28.0 (28.0, 28.0) | 26.0 (3.5, 28.0) | 28.0 (28.0, 28.0) |
| 28-day mortality *n (%)* | 1 (4%) | 1 (13%) | 4 (15%) |
| Survival free of PICU censored at 28 days *median (IQR)* | 21.8 (17.0, 25.0) | 4.3 (0.0, 13.8) | 20.0 (0.0, 25.5) |
| Length of stay in PICU *median (IQR)* | 5.1 (2.2, 7.7) | 18.3 (7.8, 29.9) | 5.3 (2.5, 11.3) |
| Length of stay in hospital *median (IQR)* | 12.2 (7.8, 22.9) | 41.5 (24.5, 61.7) | 13.6 (6.1, 29.0) |
| POPC at 28 days *median (IQR)* | 2 (1, 5) | 5 (3, 5) | 3 (2, 4) |
| Change in POPC from baseline *median (IQR)* | 0 (0, 0) | 2 (1, 3) | 1 (0, 2) |
| Functional Status Score at 28 days *median (IQR)* | 6 (6, 12) | 12 (8, 15) | 9 (7, 13) |
| Change in Functional Status Score from baseline *median (IQR)* | 0 (0, 0) | 4 (0, 9) | 2 (0, 6) |
| **Proxy measures of intervention efficacy** |  |  |  |
| Lactate <2mmol/l by 6 hours post enrolment *n (%)* | 20 (80%) | 4 (50%) | 16 (59%) |
| Lactate <2mmol/l by 12 hours post enrolment *n (%)* | 21 (84%) | 5 (63%) | 19 (70%) |
| Lactate <2mmol/l by 24 hours post enrolment *n (%)* | 22 (88%) | 6 (75%) | 22 (81%) |
| Time to reversal of tachycardia censored at 24 hours [hours] *median (IQR)* | 0.0 (0.0, 6.0) | 0.0 (0.0, 9.0) | 0.0 (0.0, 6.0) |
| Time to shock reversal censored at 28 days [hours] *median (IQR)* | 33.3 (17.3, 56.0) | 148.4 (80.8, 239.8) | 35.2 (14.6, 101.2) |

IQR, interquartile range; PICU, paediatric intensive care unit; POPC Pediatric Overall Performance Category

^*^ as measured by pediatric Sequential Organ Failure Assessment (pSOFA) score

^**^multi-organ dysfunction is defined as >1 organ with a pSOFA subscore of >0

**Table S4. Primary and secondary clinical outcomes per sensitivity analysis for subgroup of patients with septic shock and acute lung injury (ALI).** ALI was defined as study patients with a pediatric Sequential Organ Failure Assessment (pSOFA) respiratory subscore of ≥2 at randomization.

| **Outcome** | **Standard Care**  **N=13** | **Vitamin C, Hydrocortisone, and Thiamine**  **N=14** |
| --- | --- | --- |
| **Primary clinical outcome** |  |  |
| Survival free of organ dysfunction^*^ censored at 28 days *median (IQR)* | 15.0 (4.0, 23.0) | 17.0 (0.0, 20.0) |
| **Secondary clinical outcomes** |  |  |
| Survival free of inotropic support at 7 days *n (%)* | 6.1 (0.5, 7.0) | 6.4 (1.3, 7.0) |
| Survival free of multiorgan dysfunction^**^ at 7 days *n (%)* | 0.0 (0.0, 2.0) | 0.0 (0.0, 3.0) |
| Survival free of AKI at 28 days *median (IQR)* | 28.0 (7.0, 28.0) | 28.0 (24.0, 28.0) |
| 28-day mortality *n (%)* | 2 (15%) | 2 (14%) |
| Survival free of PICU censored at 28 days *median (IQR)* | 15.2 (2.5, 20.3) | 15.1 (0.0, 20.1) |
| Length of stay in PICU *median (IQR)* | 7.7 (6.2, 16.8) | 8.6 (4.8, 23.2) |
| Length of stay in hospital *median (IQR)* | 24.8 (14.3, 33.3) | 17.8 (7.6, 65.9) |
| POPC at 28 days *median (IQR)* | 4 (2, 5) | 3 (2, 4) |
| Change in POPC from baseline *median (IQR)* | 0 (0, 1) | 1 (0, 3) |
| Functional Status Score at 28 days *median (IQR)* | 8 (7, 15) | 10 (7, 14) |
| Change in Functional Status Score from baseline *median (IQR)* | 1 (0, 2) | 3 (1, 7) |
| **Proxy measures of intervention efficacy** |  |  |
| Lactate <2mmol/l by 6 hours post enrolment *n (%)* | 9 (69%) | 10 (71%) |
| Lactate <2mmol/l by 12 hours post enrolment *n (%)* | 10 (77%) | 11 (79%) |
| Lactate <2mmol/l by 24 hours post enrolment *n (%)* | 11 (85%) | 12 (86%) |
| Time to reversal of tachycardia censored at 24 hours [hours] *median (IQR)* | 0.0 (0.0, 6.0) | 0.0 (0.0, 5.7) |
| Time to shock reversal censored at 28 days [hours] *median (IQR)* | 54.8 (44.2, 169.3) | 52.6 (19.3, 122.3) |

IQR, interquartile range; PICU, paediatric intensive care unit; POPC Pediatric Overall Performance Category

^*^ as measured by pediatric Sequential Organ Failure Assessment (pSOFA) score

^**^multi-organ dysfunction is defined as >1 organ with a pSOFA subscore of >0

**Figure S1. CONSORT participant flow diagram for the RESPOND PICU trial.**

**
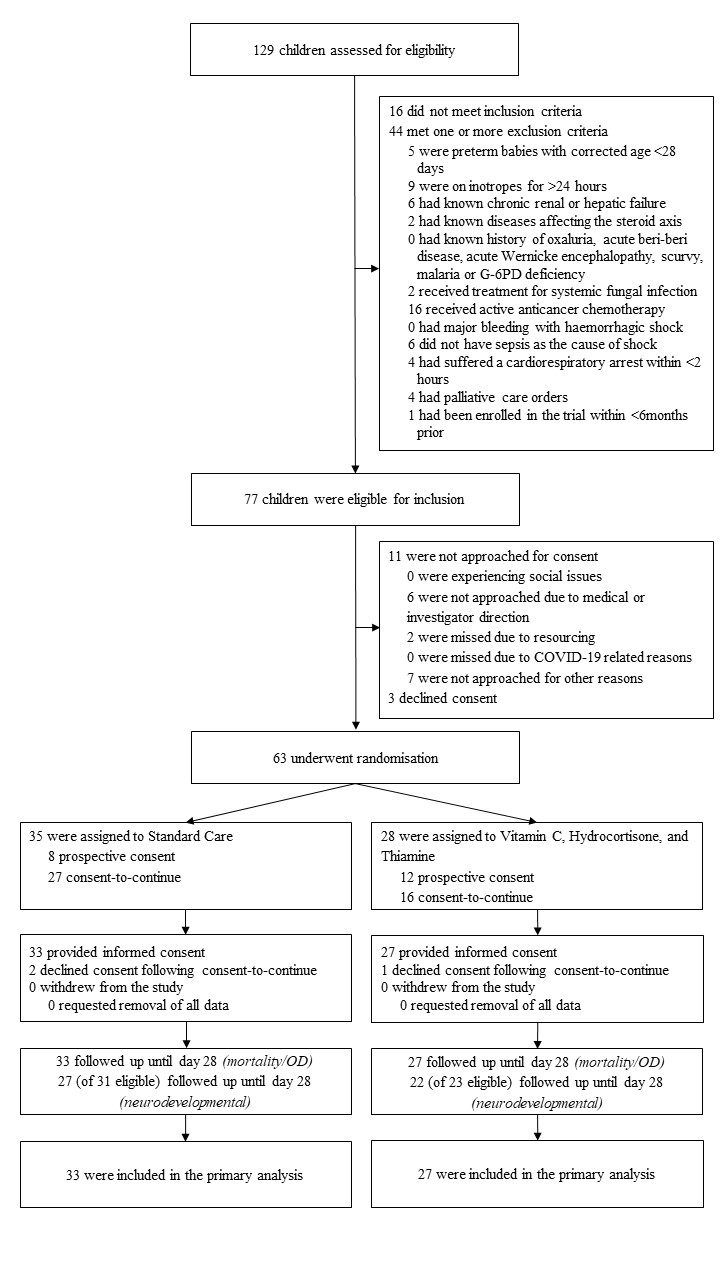
**

**Figure S2. Pediatric Overall Performance Category (POPC, Panel A, C) and Functional Status Score (FSS, Panel B) are compared between baseline (at randomization) and 28-days after randomization for patients in the standard care, versus patients in the intervention group.** POPC was reported as a 6-item score (good overall performance; mild overall disability; moderate overall disability; severe overall disability; coma or vegetative state; brain death), as shown in Figure S1C. POPC (values 1 to 6) and FSS (value 6 to 30) indicate better performance with lower values, and worse performance (i.e. disabilities) with higher values.

**A) B)**

**C)**

**Figure S3.** **Composite figure of a) shock index (heart rate by systolic blood pressure), b) heart rate, c) systolic blood pressure, d) Vasoactive Inotrope Score, e) lactate levels, and f) fluid bolus volume administered (in ml/kg) shown at 0, 1, 6, 12, and 24 hours after randomisation, separated by treatment group.**

**Figure S4.** **Cumulative incidence functions for survival free of organ dysfunction (accounting for mortality) per sensitivity analysis comparing children with the intervention (vitamin C, thiamine, hydrocortisone), with children in the standard arm treated with hydrocortisone alone, and without hydrocortisone.**

**Table S5. Protocol violations and major adverse events.**

| **Variable** | **Standard Care**  **N=33** | **Vitamin C, Hydrocortisone, and Thiamine**  **N=27** |
| --- | --- | --- |
| **Protocol violations** | N=8 | N=7 |
| Number of patients with a protocol deviation *n (%)* | 7/33 (21%) | 5/27 (19%) |
| Consultant or investigator initiated withdrawal from the study prior to the finalisation of informed consent *n (%)* | 0 | 0 |
| Time taken to obtain written informed consent exceeded 72 hours *n (%)* | 1 (13%) | 0 (0%) |
| Written informed consent obtained but no study data collected *n (%)* | 0 | 0 |
| Patient randomised but did not meet study specified inclusion/exclusion criteria for enrolment in the study *n (%)* | 2 (25%) | 2 (29%) |
| Patient randomised to an incorrect strata *n (%)* | 0 | 0 |
| Patient randomised but did not receive/commence on any study treatment(s) *n (%)* | 0 | 1 (14%) |
| First treatment received/commenced is not the same as the randomised allocation *n (%)* | 0 | 0 |
| Study treatment not delivered according to protocol *n (%)* | 1 (13%) | 4 (57%) |
| Other | 4 (50% | 0 (0%) |
| **Major adverse events** | N=10 | N=12 |
| Number of patients with an adverse event *n (%)* | 8/33 (24%) | 9/27 (33%) |
| Death *n (%) in-hospital (note: deaths occurring after 28 days were considered as well)* | 4 (40%) | 5 (42%) |
| Cardiopulmonary arrest *n (%)* | 1 (10%) | 0 |
| Extracorporeal membrane oxygenation *n (%)* | 2 (20%) | 1 (8%) |
| Amputation *n (%)* | 0 | 1 (8%) |
| Limb ischemia *n (%)* | 1 (10%) | 2 (17%) |
| Extravasation injury *n (%)* | 0 | 0 |
| Hypertension *n (%)* | 0 | 0 |
| Arrhythmia *n (%)* | 0 | 1 (8%) |
| Hyperglycemia *n (%)* | 0 | 0 |
| Abdominal compartment syndrome *n (%)* | 0 | 0 |
| Pulmonary oedema *n (%)* | 0 | 0 |
| Confirmed hospital-acquired infection *n (%)* | 0 | 0 |
| Other *n (%)* | 2 (20%) | 2 (17%) |
| Relatedness of the AE with the study intervention |  |  |
| Not related *n (%)* | 10 (100%) | 10 (83%) |
| Unlikely *n (%)* | 0 | 1 (8%) |
| Possibly *n (%)* | 0 | 0 |
| Probably *n (%)* | 0 | 0 |
| Definitely *n (%)* | 0 | 1 (8%) |

**Figure S5. Clinical course in a study patient receiving a dose of 15 g total vitamin C. The figure shows the time course since randomisation in hours until the discharge from PICU.** Inotrope duration, vasopressor requirement (blue dots), organ dysfunction scores (pink dots), and vitamin C doses administered (orange circles) are shown. pSOFA, pediatric Sequential Organ Failure Assessment score; VIS, vasopressor-inotrope score.


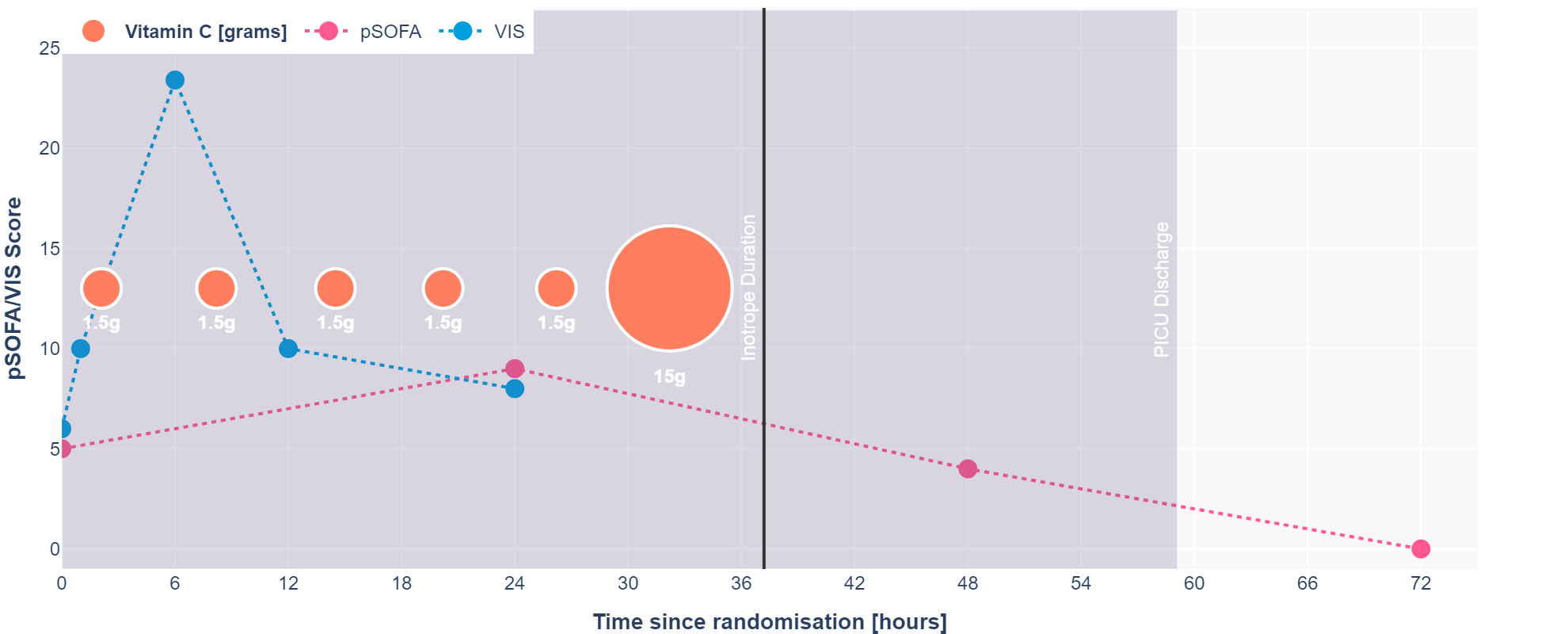

Supplement: Supplementary file 1 [file pcc-25-159-s001.docx]
